# Radiographical Assessment of Injectable Calcium Phosphate Bone Cement (Osteopaste) in Critical Size Bone Defects of Rabbit’s Tibia Model

**DOI:** 10.5704/MOJ.2507.005

**Published:** 2025-07

**Authors:** CNZ Che-Seman, Z Zakaria, Z Buyong, AR Md-Ralib, MA Sharifudin, NH Mohd-Jan

**Affiliations:** 1 Kulliyyah of Science, International Islamic University Malaysia, Kuantan, Malaysia; 2 Department of Orthopaedics, Traumatology and Rehabilitation, International Islamic University Malaysia, Kuantan, Malaysia; 3 Department of Basic Medical Sciences, International Islamic University Malaysia, Kuantan, Malaysia; 4 Department of Radiology, International Islamic University Malaysia, Kuantan, Malaysia; 5 Department of Orthopaedics, Universiti Sultan Zainal Abidin, Kuala Terengganu, Malaysia

**Keywords:** critical size defect, calcium phosphate, calcium sulphate, bone formation, osteointegration

## Abstract

**Introduction:**

Recent advances in orthopaedic research focus on improving bone healing and grafting. Osteopaste, a synthetic bone cement made from tetra-calcium phosphate (TTCP) and tri-calcium phosphate (TCP) has been developed to overcome limitations of traditional bone grafts. This study evaluates the radiographic density and new bone formation to bridge the critical size defect of Osteopaste compared to two other synthetic grafts, JectOS (calcium phosphate) and MIIG-X3 (calcium sulfate) at 6, 12, and 24 weeks.

**Materials and Methods:**

A critical size defect measuring approximately 4.5mm (width) x 9.0mm (length) was surgically created at the proximal tibial metaphysis and implanted with Osteopaste, JectOS, or MIIG-X3. Following cement implantation, surrounding soft tissues were repositioned and sutured with bioabsorbable surgical suture. Bone defect healing and cement density were qualitatively and quantitatively evaluated using plain radiographs and computed tomography (CT) scans at 6, 12, and 24 weeks.

**Results:**

The Osteopaste group showed radiographic density levels between those of JectOS and MIIG-X3. JectOS had the highest density, while Osteopaste was higher than MIIG-X3. In the Osteopaste group, new bone formation bridged the critical size defect by 12 weeks, but no bridging occurred in the other two groups at any time point. Statistical analysis showed significant differences in mean density among the groups at 6, 12, and 24 weeks (P<0.0001).

**Conclusion:**

Osteopaste effectively promotes new bone formation. Its performance falls between that of JectOS, which has the highest density, and MIIG-X3. These results suggest that Osteopaste could be a useful alternative for bone grafting.

## Introduction

The field of orthopaedics faces persistent challenges in achieving optimal bone healing. While autogenous and allogenous bone grafts are commonly utilised, they are hindered by limitations such as material availability and potential risks of infection or donor site morbidity. As an alternative, calcium phosphate cement has emerged as a promising bone graft material due to its close resemblance to natural bone in terms of calcium or phosphorus ratios and its stability in physiological environments^1^. In fact, the mineral composition of calcium phosphate, containing calcium ions (Ca^2+^), orthophosphates (PO43), metaphosphates or pyrophosphates (P2O74) and occasionally hydrogen or hydroxide ions closely mirrors that of bone^2^.

Extensively studied as a potential material for bone tissue engineering, calcium phosphate cement offers several advantages, including its ability to be directly injected into bone defects and allowed to set *in situ*^1^. Widely employed in various dental and craniofacial procedures, including frontal sinus reconstruction, augmentation of craniofacial skeletal defects, endodontics, and repair of periodontal bone and tooth defects^3,4^.

Calcium phosphates have garnered significant interest in hard tissue applications due to their bioactivity, biocompatibility, biodegradability, osteoconductive properties, osteophilic nature, and high mechanical strength. Moreover, they are non-toxic, capable of filling bone cavities, non-mutagenic, non-inflammatory, and not recognised as foreign to the body^5^. Crucially, these biomaterials exhibit bioactive behaviour, integrating into the tissue through processes akin to those active in remodelling healthy bone^6^. Numerous studies have demonstrated favourable interactions between calcium phosphate materials and living tissue, including the differentiation of immature cells into bone cells^7,8^. These materials form chemical bonds with bone along the interface, facilitated by the adsorption of bone growth-mediating proteins at the biomaterial surface, providing biochemically mediated strong bonding for osteogenesis. Furthermore, they are resorbed by osteoclasts *in vitro*, support osteoblast adhesion and proliferation, and undergo remodelling into the bone *in vivo*^1,2^.

In Malaysia, a collaborative project has successfully developed an injectable calcium phosphate known as Osteopaste (OP). This product, derived from limestone and comprising a combination of tetra-calcium phosphate (TTCP) and tri-calcium phosphate (TCP) powders, aims to compare bone graft material density and new bone formation bridging between defect borders using radiographic assessment between groups implanted with Osteopaste and commercialised bone grafts, namely JectOS (calcium phosphate) and MIIG-X3 (calcium sulphate), at different assessment periods.

## Materials and Methods

Osteopaste was prepared by mixing 3g of Osteopaste powder with 2mL of sterile distilled water in a bowl. The mixture underwent vigorous stirring with a spatula until achieving a smooth, homogenous paste consistency. Subsequently, the paste was transferred into a 10mL syringe, becoming ready for application within 4 to 6 minutes.

JectOS [Kasios, France] paste was prepared by pouring 2.5mL of JectOS liquid into a bowl, followed by the addition of 5g of JectOS powder. Vigorous mixing with a spatula ensued until the mixture transformed into a smooth, homogenous liquid, typically within approximately 30 seconds. The resulting mixture was then transferred into a 10mL syringe, ready for use after 2 to 3 minutes when it attained a paste-like consistency.

For the Minimally Invasive Injectable Graft (MIIG-X3) from Wright, USA, 1.5mL of MIIG-X3 liquid was poured into a bowl, to which 6g of MIIG-X3 powder was added. The combination underwent vigorous mixing with a spatula until it achieved a smooth, homogenous liquid consistency, typically within about 30 seconds. The resulting mixture was then transferred into a 10mL syringe, becoming ready for application after approximately 2 to 2 minutes and 30 seconds when it transformed into a paste-like form.

Approval for the animal study was obtained from the Institutional Animal Care and Use Committee of the International Islamic University Malaysia (IACUC-IIUM). Thirty-six skeletally mature New Zealand white rabbits *(Oryctolagus cuniculus)*, weighing between 2.5 kg to 3.5 kg were selected for this investigation. Upon arrival at the university animal facility, the rabbits underwent a two-week acclimatisation period. They were housed in controlled environmental conditions, maintaining a room temperature of 20°C to 25°C and humidity levels of 65% to 85%, under a 12-hour light-dark cycle. The rabbits were provided with commercial rabbit pellets and supplemented with fresh vegetables, with access to fresh water via water bottles, changed daily. Pellets and water were available ad libitum.

The rabbits were randomly assigned for grouping. The animals were divided into three groups based on the type of implant: Osteopaste, JectOS, and MIIG-X3. Each group was further subdivided into three subgroups according to the assessment periods at 6, 12, and 24 weeks, with four rabbits in each subgroup.

All animals underwent a surgical procedure to create a critical size defect in the proximal tibial metaphysis using a previously established approach technique^9^. Preparation of Osteopaste, JectOS, or MIIG-X3 occurred before implantation, following the surgical procedure to create critical size defect. Approximately 1cc of cement paste, either Osteopaste, JectOS, or MIIG-X3 was injected into the defect and placed using a McDonald elevator. Following implantation, the surgical incision was closed using a bioabsorbable surgical suture [Monosyn® 4.0, Germany], employing a continuous suturing technique for the muscle and double-layer closure with an interrupted suturing technique for the skin incision. Povidone-soaked gauze and bandages were applied for wound dressing. Enrofloxacin [10mg/kg; Batril® 5%, Bayer AG, Leverkusen, Germany] and analgesic tramadol hydrochloride [50mg/mL; Mabron, Medochemie LTD, Limassol-Cyprus] were administered daily for up to seven days post-implantation via the intramuscular route to prevent wound infection and manage pain. Plain radiographs and CT scans were conducted at 6, 12, and 24 weeks post-surgery for assessment purposes.

Radiographic evaluation was conducted at intervals of either 6, 12, or 24 weeks utilising two distinct modalities. The first modality employed was plain radiography, aimed at assessing implant performance and monitoring wound healing progress. The second modality involved computed tomography (CT) scans, utilised to ascertain the potential degradation behaviour of the implant materials within the surrounding tissue and to evaluate the presence of new bone formation bridging the critical size defect.

Prior to the procedure, rabbits received intramuscular anaesthesia using a Ketamine-Xylazine-Tiletamine/Zolazepam (KTX) mixture, as described by Che Seman *et al*^9^, and were positioned in the prone orientation. Plain radiographs were captured with anteroposterior and lateral views at 6, 12, or 24 weeks post-operatively. The radiographic images were obtained under consistent conditions with a tube-to-plate distance of 70cm, utilising 63 kilovolts (kV), 2.00 milliamperes (mAs) and 2.00 milliseconds (ms). The computed radiography system [Phillips Optimus 80, Philips Medical System, USA] utilised was equipped with a high-speed image reader [Regius 190, Konica Minolta] and laser printer [Drypro 793, Konica Minolta]. The presence of new bone formation was determined by observing bone bridging within the critical size defect area.

Animals received anaesthesia via intramuscular injection of the KTX mixture and were positioned in the supine orientation. Scanning was conducted using a compact fan beam-type tomography system. The acquired data were exported to DICOM format and re-sliced into sagittal and coronal views using OsiriX, an open-source image processing application software for DICOM files. Evaluation of new bone formation relied on identifying continuous bone bridging within the critical size defect in axial images.

**Fig. 1:**
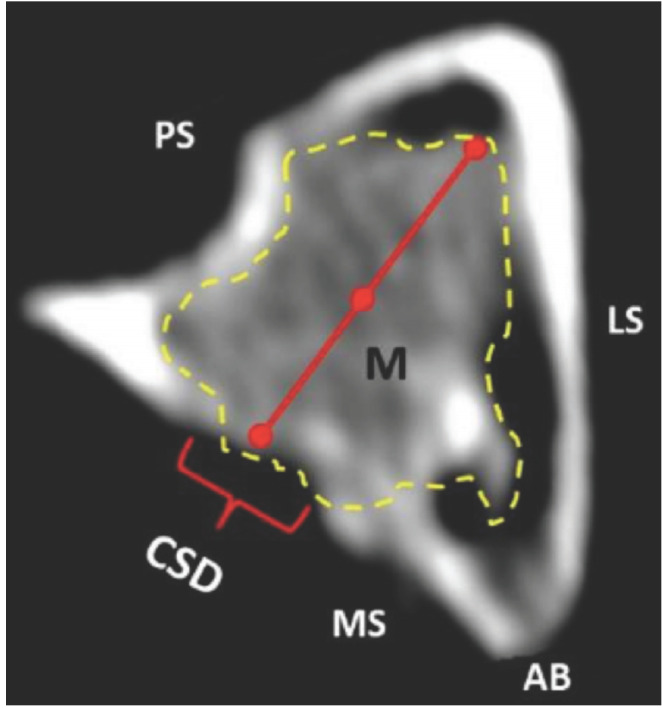
Axial view of proximal tibial of rabbit implanted with biomaterial. ‘M’ represents the area of material within the dotted line at the defect. CSD: critical size defect, M: material, AB: anterior border, MS: medial surface, LS: lateral surface, PS: posterior surface.

Axial views of CT scan images of the proximal tibia, specifically at the level of the critical size defect and implanted material, were utilised to assess material density at various assessment periods. Three points for density readings were selected: two at the periphery of the material and one at the centre (refer to Fig. 1). Density readings were analysed using OsiriX software, followed by a calculation of the average density.

Quantitative data were analysed using IBM SPSS version 24 for Windows [IBM Corp. and SPSS Inc., Chicago, USA]. Density data for Osteopaste, JectOS and MIIG-X3 were presented as mean ± standard deviation (SD). One-way ANOVA was employed to compare the mean densities of Osteopaste, JectOS, and MIIG-X3 at 6, 12, and 24 weeks. A significance level of P<0.05 was considered statistically significant.

## Results

**Fig. 2:**
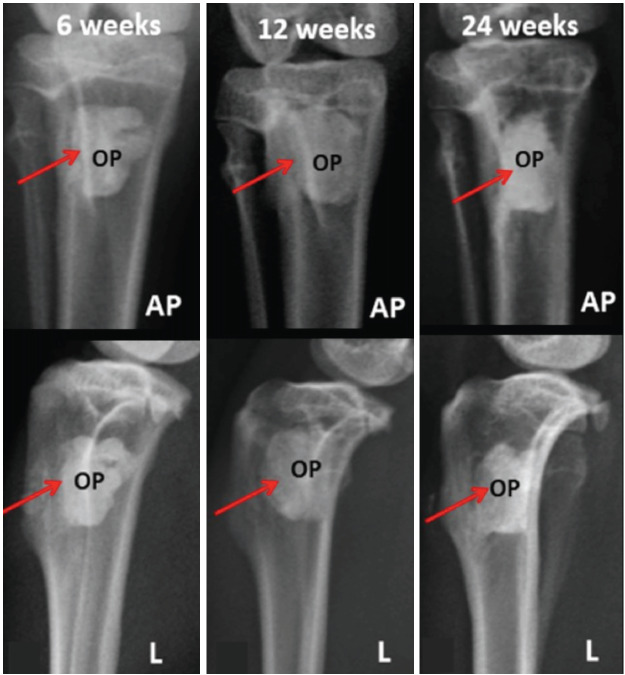
Radiographs of representative samples in Osteopaste groups at 6, 12 and 24 weeks. Red arrow: critical size defect area, OP: osteopaste, AP: anteroposterior view, L: lateral view.

In the anteroposterior and lateral views of the proximal tibia captured at 6, 12, and 24 weeks post-surgery (Fig. 2), irregular shapes were observed at the edges of the defects filled with Osteopaste. Osteopaste exhibited a radiopacity similar to that of the surrounding cortical bone. However, within the trabecular bone and bone marrow, Osteopaste could still be distinguished from the surrounding bone cortex.

**Fig. 3:**
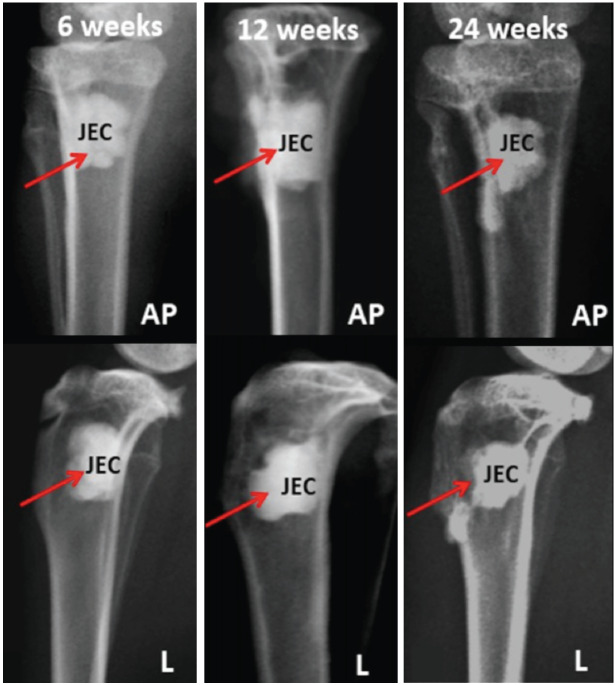
Radiographs of representative samples in JectOS groups at 6, 12 and 24 weeks. Red arrow: critical size defect area, JEC: JectOS, AP: anteroposterior view, L: lateral view.

In the radiographic images of the anteroposterior and lateral views of the proximal tibia implanted with JectOS material at 6, 12, and 24 weeks, radiolucent lines were evident between JectOS and the host bone. These lines, accompanied by the irregular shape of the material (Fig. 3), highlighted JectOS's higher radiopacity compared to the adjacent cortical bone, making it easily distinguishable from the surrounding bone.

**Fig. 4:**
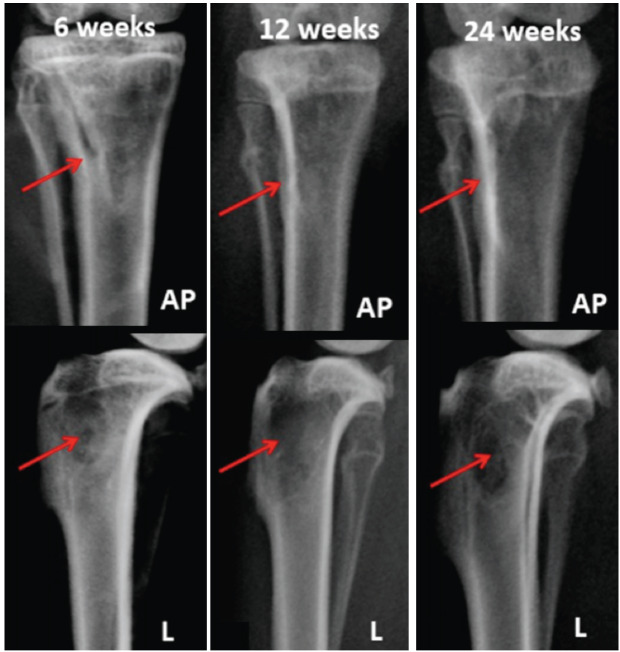
Radiographs of representative samples in MIIG-X3 groups at 6, 12 and 24 weeks. Red arrow: critical size defect area, AP: anteroposterior view, L: lateral view.

In the radiographic images of the anteroposterior and lateral views of the proximal tibia implanted with MIIG-X3 material, it was observed that MIIG-X3 was predominantly resorbed and dissolved as early as 6 weeks. Although the margins of critical size defects could still be identified on lateral views, they appeared more defined towards 24 weeks (Fig. 4).

**Fig. 5:**
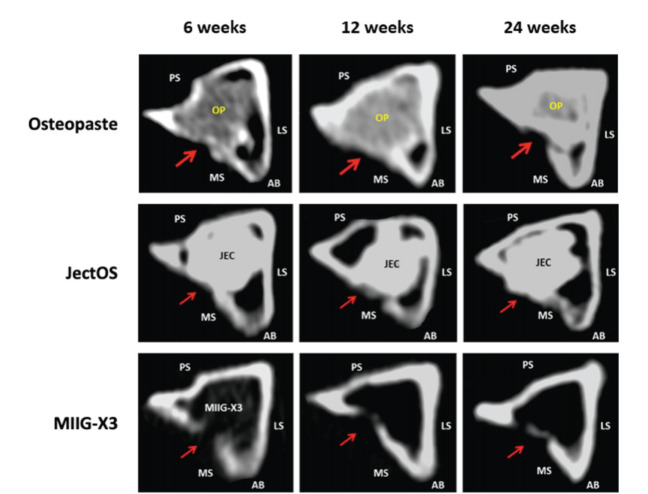
Computed tomography scans of representative sample in Osteopaste, JectOS and MIIG-X3 groups at 6, 12 and 24 weeks assessment. Red arrow: critical size defect area, AB: anterior border, MS: medial surface, LS: lateral surface, PS: posterior surface.

Representative CT scan images of the Osteopaste, JectOS, and MIIG-X3 groups at 6, 12, and 24 weeks are depicted in Fig. 5. In the axial view of the Osteopaste group at 6 weeks post-surgery, the margin of the critical size defect site was indistinct, blending with the cortical bone. This pattern persisted at 12 and 24 weeks. However, the radiopacity of the cortical bone surpassed that of Osteopaste, with clear bridging of the defect observed from 12 weeks onwards.

In the axial view of the CT scan for the JectOS group at 6 weeks post-surgery, visualisation of the critical size defect margin was hindered due to the similar density of JectOS and cortical bone. At 12 and 24 weeks post-surgery, pronounced gaps between JectOS and the critical size defect were evident, indicating the non-bridging of the defects.

For the MIIG-X3 group, the axial view of the CT scan revealed an unclear margin of the defect at six weeks. By 12 and 24 weeks, new bone formation extended towards the centre of the defect. Residual MIIG-X3 present within the critical size defect at 6 weeks displayed lower radiopacity compared to the host bone, with no residual MIIG-X3 observed at 12 and 24 weeks. Although increased new bone formation was seen, complete bridging of the defect did not occur.

**Fig. 6:**
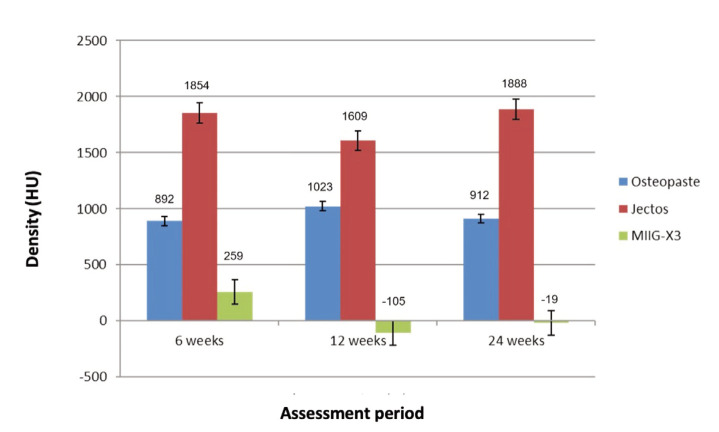
Comparison mean density between Osteopaste, JectOS and MIIG-X3 at 6, 12 and 24 weeks assessment.

Statistical analysis revealed a significant difference in mean density between the Osteopaste, JectOS, and MIIG-X3 groups at 6, 12, and 24 weeks post-surgery (P<0.0001) (Fig. 6).

Post-hoc multiple comparisons of mean density at these time points (Table I) showed that JectOS had the highest density, significantly greater than both Osteopaste and MIIG-X3, while Osteopaste demonstrated a significantly higher density than MIIG-X3 (P<0.0001). These statistical findings indicate that the JectOS group exhibited the most prominent radiographic densities at the critical size defect area over the 24 weeks. The Osteopaste group showed intermediate radiographic density, falling between the JectOS and MIIG-X3 groups.

**Table I:** Post-hoc multiple comparisons of mean density between implant materials at 6, 12 and 24 weeks post-surgery.

Type of Implant Material	Mean Difference (HU)	P-value
6 weeks		
Osteopaste vs JectOS	-961.25	<0.0001*
Osteopaste vs MIIG-X3	633.41	<0.0001*
JectOS vs MIIG-X3	1594.67	<0.0001*
12 weeks		
Osteopaste vs JectOS	-586.58	<0.0001*
Osteopaste vs MIIG-X3	1127.42	<0.0001*
JectOS vs MIIG-X3	1714.00	<0.0001*
24 weeks		
Osteopaste vs JectOS	-976.50	<0.0001*
Osteopaste vs MIIG-X3	930.50	<0.0001*
JectOS vs MIIG-X3	1907.00	<0.0001*

## Discussion

Radiopacity is a critical property that allows the visualisation of bone graft materials through radiographic imaging, aiding in quality assessment^10^. Various methods have been used to evaluate radiopacity. Tanomaru-Filho *et al*^11^ employed digital radiographic images and WIXWIN-2000 software for assessment, while Katz *et al*^12^ used an aluminium step wedge and photo densitometer to evaluate the radiopacity of gutta-percha cones. Pekkan *et al*^13^ compared the radiopacity of bone graft materials with bovine mandibular cortical bone and human dentine using a transmission densitometer. In this study, OsiriX software was used to assess the radiopacity of bone graft materials due to its established reliability and reproducibility in medical imaging analysis. OsiriX is widely used for radiological evaluations and provides advanced 3D visualisation as well as precise density measurement capabilities. Its user-friendly tools for quantitative assessments, along with compatibility with various imaging modalities, made it ideal for this study by ensuring accurate and consistent results.

Osteopaste and JectOS, both calcium phosphate-based materials, exhibit distinct radiopacity characteristics. While Osteopaste has a radiopacity similar to natural bone, JectOS shows higher radiopacity due to the inclusion of inorganic contrast agents like barium sulphate (BaSO4). This difference in radiopacity reflects the varying formulations of these materials. Osteopaste's similarity to natural bone aids integration during healing but results in lower contrast on imaging, making it difficult to distinguish from native bone tissue. In contrast, JectOS is designed to be more radiodense, enhancing visibility in radiological assessments^13,14^.

To overcome the challenge of low radiopacity, it is suggested that Osteopaste incorporate contrast agents to enhance imaging clarity. Both organic contrast agents (e.g., organo-bismuth and organo-iodine) and inorganic agents (e.g., barium sulfate (BaSO4), zirconium dioxide (ZrO2) and iron oxide nanoparticles) are commonly used to improve radiographic contrast^15-17^. Ginebra *et al*^18^ compared the effects of organic and inorganic contrast agents on material properties, finding that the addition of ZrO2 significantly improved tensile strength, fracture toughness, and resistance to fatigue crack propagation. In contrast, adding BaSO4 decreased tensile strength but did not affect fracture toughness while enhancing crack propagation resistance. Meanwhile, the addition of iodine containing monomers increased tensile strength and fracture toughness but did not improve fatigue crack propagation resistance.

MIIG-X3 is a calcium sulphate-based bone graft substitute and typically exhibits lower radiopacity. A radiopaque contrast agent, such as BaSO4 or ZrO2 may be added to the formulation to enhance its visibility in radiographic imaging. These agents improve the material's contrast, making it easier to distinguish from surrounding tissues in imaging studies. By incorporating such radiopaque additives, MIIG-X3 can be more effectively monitored for integration and bone regeneration during post-operative assessments^19^. In this study, the residual MIIG-X3 had lower radiopacity than the host bone at six weeks, likely due to its rapid resorption. Kim *et al*^20^ reported that MIIG-X3’s resorption can still be monitored radiographically, but the rapid degradation poses a challenge in maintaining scaffold support until full bone regeneration is achieved.

Johnson *et al*^21^ emphasised that the rate of resorption of radiodense material intended for use as a bone substitute should be rapid enough to allow radiographic evaluation. This is crucial for distinguishing between the healing process of bone and residual radiodense material, preventing the masking of non-union by the presence of radiodense material. Complete resorption over time also mitigates potential complications associated with long-term implantation of foreign bodies. Low-density materials may result in poor quality radiograph images, posing challenges for immediate and short-term post-operative monitoring of bone formation by clinicians^15^.

The radiographic density observed in the critical size defect varied across materials, with JectOS showing the highest density at all assessment points, followed by Osteopaste and MIIG-X3. This suggests that Osteopaste’s degradation rate is slower than MIIG-X3 but faster than JectOS. Minimal resorption in Osteopaste and JectOS allowed for sustained structural support, which facilitated bone formation and defect bridging over 24 weeks. In particular, the critical size defect was fully bridged in the Osteopaste group by 12 weeks, indicating its effective integration and support for bone regeneration. Osteopaste’s degradation increased the surface area within the defect, promoting greater ion release, apatite formation and subsequent bone growth^22,23^. However, the slower degradation of JectOS, while maintaining structural support also slowed its resorption and delayed bone healing, as the critical size defect in the JectOS group remained unbridged throughout the assessment period.

In contrast, MIIG-X3 exhibited complete resorption by 12 weeks. The faster resorption rate of MIIG-X3 compared to calcium phosphate cements like Osteopaste and JectOs is due to its composition, which allows for quicker degradation and remodeling in the body. Although rapid resorption can accelerate initial healing, it can also result in the premature loss of scaffold support before complete bone regeneration. This can lead to fibrous tissue infiltrating the defect site, which compromises full bone consolidation, as bone requires a longer period to mature and fully integrate within the defect. This finding is supported by the result of Kumar *et al*^19^. As contradicts to previous study by Kim *et al*^20^ that found calcium sulphate failed to bridge the defect due to cyst formation and poor blood supply to this defect. An ideal bone graft material should balance resorption with gradual new bone infiltration, allowing for effective and sustained bone regeneration over time.

The main limitation of this study is the lack of information on the percentage of new bone formation across the Osteopaste, JectOS and MIIG-X3 groups at all assessment periods. Histological analysis is essential to measure and compare the percentage of new bone formation among these implant materials through histomorphometric data. Additionally, histological assessment is generally more accurate than radiographic methods for tissue examination^24^.

## Conclusion

The study found that the Osteopaste group had radiographic density levels between those of the JectOS and MIIG-X3 groups. New bone bridging in the Osteopaste group was observed by 12 weeks, while no bridging occurred in the JectOS and MIIG-X3 groups during any assessment period. These results indicate that Osteopaste acts as a biodegradable material for bone repair, performing better than JectOS and MIIG-X3.
